# Risk Behaviors and Reasons for not Getting Tested for HIV among Men Who Have Sex with Men: An Online Survey in Peru

**DOI:** 10.1371/journal.pone.0027334

**Published:** 2011-11-09

**Authors:** Magaly M. Blas, Isaac E. Alva, Robinson Cabello, Cesar Carcamo, Ann E. Kurth

**Affiliations:** 1 Epidemiology, HIV and STD Unit, School of Public Health and Administration, Universidad Peruana Cayetano Heredia, Lima, Peru; 2 NGO Via Libre, Lima, Peru; 3 Department of Global Health, School of Nursing, University of Washington, Seattle, Washington, United States of America; UCL Institute of Child Health, University College London, United Kingdom

## Abstract

**Background:**

Men who have sex with men (MSM) account for the greatest burden of the HIV epidemic in Peru. Given that MSM are frequent users of the Internet, understanding the risk behaviors and the reasons for not getting tested among MSM who surf the Internet may improve the tailoring of future online behavioral interventions.

**Methods:**

From October 2007 to April 2008, we conducted an online survey among users of seven Peruvian gay websites.

**Results:**

We received 1,481 surveys, 1,301 of which were included in the analysis. The median age of the participants was 22.5 years (range 12 – 71), 67% were homosexual, and the remainder was bisexual. Of survey respondents, 49.4% had never been tested for HIV and only 11.3% were contacted in-person during the last year by peer health educators from the Peruvian Ministry of Health and NGOs. Additionally, 50.8% had unprotected anal or vaginal sex at last intercourse, and a significant percentage reported a condom broken (22.1%), slipped (16.4%) or sexual intercourse initiated without wearing a condom (39.1%). The most common reasons for not getting tested for HIV among high-risk MSM were “I fear the consequences of a positive test result” (n = 55, 34.4%), and “I don't know where I can get tested” (n = 50, 31.3%).

**Conclusions:**

A small percentage of Peruvian MSM who answered our online survey, were reached by traditional peer-based education programs. Given that among high-risk MSM, fear of a positive test result and lack of awareness of places where to get tested are the most important reasons for not taking an HIV test, Internet interventions aimed at motivating HIV testing should work to reduce fear of testing and increase awareness of places that offer free HIV testing services to MSM.

## Introduction

Although the HIV epidemic in Latin America remains generally stable, HIV transmission continues to occur among persons practicing high risk behaviors such as male sex workers and men who have sex with men (MSM), in particular those with limited access to HIV prevention interventions and HIV treatment, care, and support services [1], [2].

In Peru, sex between men continues to represent the main route of HIV transmission; [3] the average HIV prevalence among MSM from high prevalence cities is 12.4%, with a prevalence in the capital city of Lima reaching as high as 22.3% [4], [5]. These HIV prevalence levels are substantially higher compared with the 0.4% prevalence in the general population [3]. The prevalence of other sexually transmitted infections (STIs) among Peruvian MSM is also high: 12.4% for syphilis and 46.3% for herpes simplex virus type 2 [4], [6].

In Peru, interventions funded by the government and by external sources are based solely on peer education and are not exploring alternative ways to reach MSM, such as online interventions [7]. Internet interventions in Peru have proven to access high-risk MSM who are interested in receiving web-based HIV prevention interventions [7], [8]. An online video-based intervention has been effective in motivating behavior change, increasing HIV testing among non-gay-identified MSM [9].

Understanding the profile of MSM who use the Internet may improve the tailoring of future online behavioral interventions. The aims of this study were to collect demographic characteristics, sexual and non-sexual risk behaviors for HIV and STIs, as well as reasons MSM who visit Peruvian gay websites have for not getting tested for HIV.

## Methods

From October 2007 to April 2008, we conducted a cross-sectional study whose target population was MSM who visited any of seven Peruvian gay Websites: http://www.gayperu.com, http://peruesgay.com, http://diariodelimagay.com, http://deambiente.com, http://chicoslima.com, http://mhol.org.pe and http://runa.org.pe, The first five websites were commercial gay websites and the last two were advocacy gay websites. We advertised animated banner ads that, if clicked, redirected the participants to our study website.

Our website included a homepage with information about the objective of the study, the content of the questionnaire, the optional nature of all the questions, and the length of the survey. It also included information about risks and benefits of participation, and privacy policy information (we mentioned that we were not going to collect information that could potentially identify participants and suggestions about how participants could prevent unauthorized access to their survey). The website also included our email and phone number for the participants to call if they needed more information. Participants had the alternative to click in a button labeled “I want to participate” to fill out the online survey, or click on “ I don't want to participate,” in which case they were asked about reasons for not wanting to participate. We did not offer any incentive for study participation. The contents of the questionnaire were available in a separate link to anyone visiting the website, regardless of their intention to participate, thus preventing people from entering the data collection form only to explore its contents.

The online survey was an open survey designed using limesurvey [10], an open source tool that allows branching, recovery of partially completed questionnaires, and frontend and backend in Spanish. We piloted the survey for language, workflow, and accurate interpretation of question meaning with 20 participants before its launching.

The survey documented demographic characteristics, sexual self-perception (how the participant identified himself, e.g. gay, bisexual, heterosexual), sexual orientation (how the participant behaved, e.g. if he had sex only with men, with women or with both), sexual role (if the participant self-identified as insertive, receptive, or versatile), access to HIV prevention interventions, sexual and non sexual risk behaviors for STIs, presence of STI symptoms, online sex seeking behavior, history of previous HIV testing and reasons for not taking an HIV test. The questionnaire design was based on previous studies on HIV and STIs conducted in Peru [7], [11], and the questions about the reasons for not getting an HIV test were based on previous studies about this topic as well as on two focus groups with each MSM subpopulation: gay and non-gay-identified MSM, and transvestites [12], [13]. Participants were able to review and change their answers before submitting the questionnaire. In the questionnaires, participants were not asked for any personally identifiable information. However, they were asked to provide an email address (one not showing personal information was preferred) to identify duplicate entries from the same individual. We did not use IP addresses to identify duplicate entries because the majority of participants in Peru answer online surveys through commercial cybercafés [7], thus it is possible to receive surveys from different participants with the same IP address within a short time period.

Data analysis was conducted using STATA 8.0 software. Chi square and Fisher's exact test were performed to assess differences in reported behaviors.

Our proposal and this study were approved by the Institutional Review Board of the University of Washington in Seattle and the non-governmental organization (NGO) Via Libre in Lima, Peru. All enrollees provided a web-based informed consent for the online questionnaire.

## Results

### Characteristics of the participants

During the five months of the study we received 1481 surveys. Of these, 180 were excluded due to the following reasons: 25 had duplicate email addresses, 85 reported a foreign residence country, 35 were women, and 35 were men who reported having sex only with women. The study participants (n = 1301) were from 24 of the 25 departments of Peru, and 81.6% (1062) of participants were from Lima. The median age of the participants was 22.5 (range 12 – 71) and the majority were in the 18–25 year age group (46.0%); 16.2% (236) of the participants were younger than 18 years old. Most of the participants had high school education or higher and answered the online survey either at home or at an Internet café (Table 1).

**Table 1:** Demographic characteristics, self-identification, and sexual orientation among MSM who answered an online survey in Peru (N = 1301).

Characteristics	N* (%)
**Median age (range)**	22.5 (12–71)
**Education**	
<High school	71 (5.6)
High school graduate	281 (22.1)
University/Technical non-graduate	446 (35.1)
University/Technical graduate	474 (37.3)
**Place of Internet access**	
Home	581 (45.3)
Internet cafes	578 (45.1)
Work	99 (7.7)
Study Center	16 (1.3)
Other	8 (0.6)
**Sexual self-perception**	
Gay	653 (50.2)
Bisexual	442 (34.0)
*Caleta* (closeted or semi-closeted)	100 (7.7)
*Hombre* (man) or heterosexual	79 (6.0)
Trans&	19 (1.5)
*Flete* (young male prostitutes)	8 (0.7)
**Sexual orientation**	
Homosexual	804 (67.0)
Bisexual	396 (33.0)

*Numbers may not add to the total because of missing data.

& Includes transvestite, transgender and transsexual.

The majority of participants declared themselves as gay, followed by bisexual, caleta (men who are closeted or semi-closeted) and hombre (man)/heterosexual. Overall, 11.3% participants received in-person HIV/STI information and 7.9% received free condoms from a peer educator from the Peruvian Ministry of Health and NGOs within the last year; 28.5% participants received in-person HIV/STI information and 12.6% received free condoms from a health-care professional during the last year (Table 1).

### Sexual and non-sexual risk behaviors for HIV/STI and HIV testing

The most common sexual role was moderno (i.e., versatile) followed by pasivo (receptive) and activo (insertive). Only 39.2% participants had a stable last sexual partner; the remaining had casual, anonymous or commercial partners. Regarding condom use at last intercourse, 50.8% had unprotected anal or vaginal sex. Table 2 shows the distribution of participants who had unprotected sex by their role on their last sexual intercourse. Most participants (61.9%) did not know the HIV status of their last sexual partner, and nearly 50% stated that they have never or almost never used a condom within the last three months. Overall, 22.1% (82) stated that they had a broken condom, 16.4% (62) that their condom slipped off, 13.7% (51) that they removed a condom during intercourse, and 39.1% that they initiated intercourse without wearing a condom within the last three months.

**Table 2:** Sexual and non-sexual risk behaviors for HIV/STI among MSM who answered an online survey in Peru (N = 1301).

Sexual Risk Behavior	N* (%)
**Sexual role**	
Activo (insertive)	224 (19.1)
Pasivo (receptive)	311 (26.5)
Moderno (versatile)	640 (54.5)
**Type of last sexual partner**	
Stable	446 (39.2)
Casual	380 (33.4)
Anonymous	256 (22.5)
Commercial	48 (4.2)
Other	9 (0.8)
**Unprotected sex at last intercourse**	
Anal insertive unprotected intercourse among those who had anal insertive sex	146 (50.2)
Anal receptive unprotected intercourse among those who had anal receptive sex	217 (49.2)
Anal insertive and receptive unprotected intercourse among those who had anal insertive and receptive sex at last intercourse	107 (55.4)
Vaginal unprotected intercourse among those who had vaginal sex at last intercourse	24 (51.1)
**HIV status of the last sexual partner**	
HIV positive	20 (1.9)
HIV negative	391 (36.3)
Unknown HIV status	667 (61.9)
**Use of condoms during the last 3 months**	
Never	235 (37.2)
Almost never	75 (11.9)
Almost always	96 (15.2)
Always	226 (35.8)
**Partner had genital ulcers or abnormal genital discharge within the last three months**	
Yes	36 (5.5)
No	614 (94.5)
**Previous HIV test**	
Never	642 (49.4)
Less than 7 months ago	272 (21.0)
7 to 11 months ago	107 (8.2)
More than 12 months ago	280 (21.5)
**Results of HIV test**	
Positive	64 (8.7)
Negative	650 (88.4)
Indeterminate	10 (1.4)
Never went to pick up results	11 (1.5)

*Numbers may not add to the total because of missing data.

Regarding STI symptoms during the last 12 months, 173 (13.3%) MSM said they had recently experienced a burning sensation during urination, 62 (4.8%) genital ulcers, 59 (4.5%) anal warts, 57 (4.4%) anal ulcers, 54 (4.2%) abnormal urethral discharge, 35 (2.7%) genital warts, and 14 (1.1%) abnormal anal discharge. Only 132 (76.3%) MSM who reported an STI symptom during the last 12 months sought a health care provider.

Nearly half of the participants had never been tested for HIV before (49.4%). Of those who tested, 41.4% tested only once and 8.7% had a positive HIV result (Table 2).

Regarding the use of the Internet during the last three months, 537 (44.0%) used it to seek information about HIV/STI and 850 (68.4%) used it to seek sexual partners. Among the participants who sought sexual partners, 365 (43.6%) also sought HIV/STI information online and among the participants who did not seek sexual partners, 168 (44.7%) sought HIV/STI information online (p = 0.76). During the last three months, 544 (44.1%) had sex with someone they met over the Internet.

Regarding drug consumption during the last sexual intercourse, 157 (12.1%) participants reported use of alcohol, 10 (0.8%) marijuana, 10 (0.8%) cocaine hydrochloride, 9 (0.7%) sildenafil, 3 (0.2%) crack cocaine, 3 (0.2%) anxiolytics, 2 (0.2%) inhalants, 2 (0.2%) ecstasy, 2 (0.2%) intravenous drugs, and 1 (0.1%) pain medications. No participant used amphetamines.

### Reasons for not getting tested for HIV

In the analysis of reasons for not getting tested for HIV we included only sexually active participants who never had an HIV positive result and who had not tested for HIV within the last year (n = 801). We divided the participants in two groups according to the type of last sexual partner and use of condoms at last sexual intercourse: The low-risk group included participants whose last sexual partner was stable (regardless of whether they used a condom or not) and participants whose last sexual partner was not stable but who used a condom in their last sexual intercourse. The high-risk group included participants who had a non-stable last sexual partner with whom they did not use a condom during their last sexual intercourse.

The most common two reasons for not getting tested for HIV among participants in the low risk group were “I fear the consequences of a positive test result” (33.6%), followed by “I always use protection” (30.8%). The most common two reasons for not getting tested among participants in the high-risk group were “I fear the consequences of a positive test result” (34.4%), followed by “I don't know where I can get tested” (31.3%).

When comparing differences in the main reasons for not getting tested for HIV among participants in the low and high-risk group, we found that participants in the low-risk group reported at a higher percentage that “they have never been at risk for infection” (27.9% vs. 20.0%, p = 0.05) and that “they always use protection” (30.8% vs. 8.8%, p<0.001). We also found that participants in the high-risk group reported at a higher percentage that “they cannot pay for the HIV test” (23.8% vs. 15.2%, p = 0.02) and that “they dońt know where to get tested” (31.3% vs. 21.1%, p = 0.01; Table 3).

**Table 3:** Comparison of the main reasons for not getting tested for HIV among MSM who had not tested for HIV within the last year, stratified by level of risk (n = 801).

	Risk level		
Reasons for not getting an HIV test	Low	High	P
	N* (%)	N* (%)	
Fear of the consequences of a positive test result	148 (33.6)	55 (34.4)	0.85
I prefer not to know if I am infected	46 (10.4)	19 (11.9)	0.61
My life won't be the same if I find out I am HIV positive	77 (17.5)	31 (19.4)	0.59
I have never been at risk for infection	123 (27.9)	32 (20.0)	0.05
I always use protection	136 (30.8)	14 (8.8)	<0.001
I am afraid that others would know that I am HIV positive	57 (12.9)	25 (15.6)	0.39
I fear the lack of confidentiality of the health care personnel	50 (11.3)	19 (11.9)	0.86
I won't get support from my family/friends/partner if I am diagnosed with HIV	53 (12.0)	18 (11.3)	0.80
I won't be able to pay for HIV treatment	65 (14.7)	26 (16.3)	0.65
I can't pay for the HIV test	67 (15.2)	38 (23.8)	0.02
I don't know where I can get tested	93 (21.1)	50 (31.3)	0.01
There is no treatment for HIV	20 (4.5)	8 (5.0)	0.81
I am afraid of blood and needles	39 (8.8)	8 (5.0)	0.12

*Numbers may not add to the total because of missing data.

& The low-risk group included participants whose last sexual partner was stable (regardless of whether they used a condom or not) and participants whose last sexual partner was not stable but who used a condom in their last sexual intercourse. The high-risk group included participants who had a non-stable last sexual partner with whom they did not use a condom during their last sexual intercourse.

## Discussion

The Internet is a suitable venue to reach at risk MSM who have not received any kind of in-person HIV-prevention interventions during the last year. Of our sample, only 11.3% received HIV/STI information from a peer educator and only 28.5% received this information from a health-care professional during the last year. For these reasons, the different institutions that conduct HIV/STI prevention activities in Peru should consider the Internet as an alternative tool to provide behavioral interventions to Peruvian MSM [7], [8].

Internet interventions have the ability to reach MSM from virtually all urban centers in Peru, as is the case with our survey. Nevertheless the majority of online surveys (81.6%) were received from Lima, a city that contains one third of the population of the country [14]. Lima is also the city that concentrates most of the HIV infections in the country and has the highest proportion of MSM who are HIV positive (22.3%) [4], [14].

Internet approaches in Peru can reach a young MSM population: the median age of our participants was 22.5 years and the majority of them were between the ages of 18 to 25. Reaching young Peruvian MSM is very important given that HIV disproportionately affects this population; of note it is estimated that the median age of HIV infection for Peruvian men is around 20 years old [14]. Another finding from our study is the ability to reach through gay websites at-risk participants younger than 18 years old­­–a population not targeted by the majority of HIV prevention programs in Peru.

However, the Internet misses less-educated MSM; the majority of our Internet participants had high school or university/technical education. It has been reported that participants with higher education are four times more likely than their less-educated counterparts to have current access to the Internet [15]. In a study conducted in China, only 78 (3.3%) of the MSM population who sent an online survey had attended junior high-school or less [16].

In contrast to studies from developed countries where Internet access is usually in private settings, one of the most common places of Internet access in our study (45.1%) was in Internet cafes (*cabinas públicas*), small-scale storefront operations that offer low-cost and reliable connections [8]. This phenomenon indicates that the *cabinas* may constitute an important venue to develop structural interventions to reach MSM in Lima [8].

The most common self-identifications from our online survey were gay (50.2%) and bisexual (34.0%). Similarly, in the 2002 sentinel surveillance in Lima, the largest proportion of participants self-identified as either homosexuals/gays (n = 562, 42.3%) or bisexuals (n = 362, 27.3%) [4]. This was most likely due to the fact that in both the online survey and the sentinel surveillance the sampling was done in gay venues. In our study, few trans (transvestites, transexuals, transgenders) and heterosexually-identified MSM sent online surveys. Regarding the ‘trans’ categories, a likely reason is the lower level of literacy that these populations have due to stigma and discrimination at school age leading to high rates of dropouts; thus the Internet may not be a useful tool to reach this population. In the case of the heterosexually-identified MSM group, they were not reached in a significant amount because this population likely visit heterosexually oriented websites as opposed to gay websites (where we advertised our study).

Through the Internet we can reach high-risk MSM population. MSM had a high percentage of unprotected sex both at last sexual intercourse and within the last three months. The majority of MSM did not know the HIV status of their last sexual partner and a substantial percentage experienced condom breakage or slippage, or initiated sexual intercourse without wearing a condom. However, participants from our study who used the Internet to engage in high-risk practices also sought HIV/STI information online. Future behavioral online interventions in Peru should include accurate HIV/STI information tailored to MSM, and they should address the correct use of condoms, as well as the importance of knowing the HIV status of the sexual partner.

The majority of MSM have not been tested for HIV during the last year (70.9%). A common reason for not getting tested was the fear of the consequences of a positive result. “Fear appeal” campaigns were popular at the beginning of the epidemic in Peru, and although there is still a debate about whether these campaigns produce safer behaviors towards HIV [17], [18], they seem to have had a counterproductive effect in motivating MSM to get tested for HIV.

Although there are programs from the Ministry of Health and NGO that provide free HIV testing at different venues in Lima [14], we found that substantial proportions of MSM at high-risk for infection state that “they don't know where to get tested” and that “they cannot pay for the HIV test” as main reasons for not getting tested. Online campaigns that increase awareness of places that offer these services for free are needed.

Our study has some limitations; first, our sample is not representative of the MSM population in Lima or Peru. Second, given that we did not collect information on view rates, and participation rates, we do not know if our sample is representative of the MSM population who visit the gay websites where we advertised our study. Third, our sampling is likely to be biased in terms of educational background, and age. Fourth, it is possible that we misclassified the participants in the low and high risk groups because we only used the last sexual partner and condom use in the last sexual intercourse. Fifth, we may have self-misrepresentation of some participants leading to misclassification (e.g., female participants answering as a male). Sixth, we may have duplicate entries from the same individual; although we excluded records for duplicate email addresses, we were not able to use cookies or IP addresses to identify potential duplicate entries from the same user. Study strengths include the fact that no financial incentives were offered for study participation.

In conclusion, a small percentage of Peruvian MSM who surf the Internet are reached by traditional peer-based education programs. Online interventions should consider the delivery of messages that address the correct use of condoms, as well as the importance of getting tested for HIV since high-risk behaviors and absence of HIV testing were common among Peruvian MSM who responded to our online survey.

Given that fear of a positive result and lack of awareness of where to get tested were the most common reasons reported for not taking an HIV test among high-risk MSM, interventions aimed at motivating HIV testing in this population should work to reduce fear of testing and increase awareness of venues that offer free HIV testing services.

## References

[1] UNAIDS (2010). UNAIDS report on the global AIDS epidemic 2010.. http://www.unaids.org/globalreport/documents/20101123_GlobalReport_full_en.pdf.

[2] UNAIDS (2007). UNAIDS policy brief: HIV and sex between men.. http://data.unaids.org/pub/BriefingNote/2006/20060801_policy_brief_msm_en.pdf.

[3] UNAIDS (2009). Epidemiological Fact Sheet on HIV and AIDS: Peru.. http://www.unaids.org/en/regionscountries/countries/peru.

[4] Sanchez J, Lama JR, Kusunoki L, Manrique H, Goicochea P (2007). HIV-1, sexually transmitted infections, and sexual behavior trends among men who have sex with men in Lima, Peru.. J Acquir Immune Defic Syndr.

[5] Goodreau SM, Peinado J, Goicochea P, Vergara J, Ojeda N (2007). Role versatility among men who have sex with men in urban Peru.. J Sex Res.

[6] Lama JR, Lucchetti A, Suarez L, Laguna-Torres VA, Guanira JV (2006). Peruvian HIV Sentinel Surveillance Working Group: Association of herpes simplex virus type 2 infection and syphilis with human immunodeficiency virus infection among men who have sex with men in Peru.. J Infect Dis.

[7] Blas MM, Alva IE, Cabello R, Garcia PJ, Carcamo C (2007). Internet as a tool to access high-risk men who have sex with men from a resource-constrained setting: a study from Peru.. Sex Transm Infect.

[8] Curioso WH, Blas MM, Nodell B, Alva IE, Kurth AE (2007). Opportunities for providing web-based interventions to prevent sexually transmitted infections in Peru.. PLoS Med.

[9] Blas MM, Alva IE, Carcamo CP, Cabello R, Goodreau SM (2010). Effect of an online video-based intervention to increase HIV testing in men who have sex with men in Peru.. PLoS One.

[10] LimeSurvey.. http://www.limesurvey.org.

[11] Garcia P, Hughes J, Carcamo C, Holmes KK (2003). Training pharmacy workers in recognition, management, and prevention of STDs: district-randomized controlled trial. Bull.. World Health Organ.

[12] Mikolajczak J, Hospers HJ, Kok G (2006). Reasons for not taking an HIV-test among untested men who have sex with men: an Internet study.. AIDS Behav.

[13] Kellerman SE, Lehman JS, Lansky A, Stevens MR, Hecht FM (2002). HIV testing within at-risk populations in the United States and the reasons for seeking or avoiding HIV testing.. J Acquir Immune Defic Syndr.

[14] Peruvian Ministry of Health (2006). http://www.portalsida.org/repos/analisis%20epidemiologica%20vih%20peru%202006.pdf.

[15] Kalichman SC, Benotsch EG, Weinhardt LS, Austin J, Luke W (2002). Internet use among people living with HIV/AIDS: association of health information, health behaviors, and health status.. AIDS Educ Prev.

[16] Zhang D, Bi P, Lv F, Tang H, Zhang J (2007). Internet use and risk behaviours: an online survey of visitors to three gay websites in China.. Sex Transm Infect.

[17] Slavin S, Batrouney C, Murphy D (2007). Fear appeals and treatment side-effects: an effective combination for HIV prevention?. AIDS Care.

[18] Green EC, Witte K (2006). Can fear arousal in public health campaigns contribute to the decline of HIV prevalence?. J Health Commun.

